# Systemic TLR2 agonist exposure regulates hematopoietic stem cells via cell-autonomous and cell-non-autonomous mechanisms

**DOI:** 10.1038/bcj.2016.45

**Published:** 2016-06-17

**Authors:** A C Herman, D A Monlish, M P Romine, S T Bhatt, S Zippel, L G Schuettpelz

**Affiliations:** 1 Department of Pediatrics, Washington University School of Medicine, St Louis, MO, USA

## Abstract

Toll-like receptor 2 (TLR2) is a member of the TLR family of receptors that play a central role in innate immunity. In addition to regulating effector immune cells, where it recognizes a wide variety of pathogen-associated and nonpathogen-associated endogenous ligands, TLR2 is expressed in hematopoietic stem cells (HSCs). Its role in HSCs, however, is not well understood. Furthermore, augmented TLR2 signaling is associated with myelodysplastic syndrome, an HSC disorder characterized by ineffective hematopoiesis and a high risk of transformation to leukemia, suggesting that aberrant signaling through this receptor may have clinically significant effects on HSCs. Herein, we show that systemic exposure of mice to a TLR2 agonist leads to an expansion of bone marrow and spleen phenotypic HSCs and progenitors, but a loss of HSC self-renewal capacity. Treatment of chimeric animals shows that these effects are largely cell non-autonomous, with a minor contribution from cell-autonomous TLR2 signaling, and are in part mediated by granulocyte colony-stimulating factor and tumor necrosis factor-α. Together, these data suggest that TLR2 ligand exposure influences HSC cycling and function via unique mechanisms from TLR4, and support an important role for TLR2 in the regulation of HSCs.

## Introduction

Toll-like receptors (TLRs) are pattern recognition receptors that play a central role in innate immunity. Recent reports suggest that TLR signaling regulates not only effector immune cells, but may also influence hematopoietic stem cells (HSCs). HSCs express multiple TLRs,^1, 2^ and *in vitro* exposure to TLR agonists stimulates HSC cycling and skews HSC differentiation toward the myeloid lineage.^1, 2, 3^ Furthermore, chronic treatment of mice with the TLR4 agonist lipopolysaccharide leads to HSC cycling and expansion, but decreased repopulating activity.^4, 5^ Together, these studies suggest that TLR signaling may shape the immune response from the level of the HSC, regulating the proliferation, differentiation and activity of these cells, and chronic exposure to TLR signals may impair HSC function.

Although accumulating reports support a role for TLR signaling in regulating HSCs, the mechanism of these effects is not clear. TLRs are expressed on many hematopoietic and stromal cell types, and therefore systemic TLR ligand exposure could have direct or indirect effects on HSCs. Indeed, proinflammatory cytokines are produced by effector immune cells and hematopoietic progenitors in response to TLR ligands,^6^ and such cytokines themselves can influence HSC cycling and function.^7^ In addition, it is not known whether HSCs respond similarly to stimulation of different TLRs. The existing literature has largely focused on TLR4, with recent studies showing that TLR4 ligation indirectly promotes HSC mobilization via production of granulocyte colony-stimulating factor (G-CSF) by endothelial cells.^8, 9^ It is not clear how stimulation of other TLR family members affects HSCs, and whether they similarly promote G-CSF-mediated HSC mobilization.

In this study, we sought to elucidate the effects of systemic TLR2 ligand exposure on HSCs and determine the cell-autonomous versus non-autonomous effects of such exposure. We specifically focused on TLR2 signaling, as although TLR2 is expressed on HSCs,^1, 10^ its role in regulating these cells is not clear. Furthermore, multiple studies have identified increased TLR expression and signaling (particularly TLR2) in the CD34+ cells of patients with myelodysplastic syndromes (MDS), a group of hematopoietic stem cell disorders associated with ineffective hematopoiesis and a high risk of leukemic transformation.^11, 12, 13, 14, 15^ This fact supports the idea that TLR2 may regulate HSCs, and aberrant signaling through this receptor may have clinically significant effects on HSCs. In fact, a clinical trial using a TLR2 antagonist for the treatment of patients with MDS is currently underway (Opsona Therapeutics, Dublin, Ireland), and thus an understanding of the role of TLR2 signaling in regulating HSCs is highly relevant toward advancing therapy for patients with MDS.

We and others previously reported that TLR2 is not necessary for HSC function (in fact, loss of TLR2 leads to improved bone marrow repopulating activity).^16, 17^ Herein, we show that augmented TLR2 signaling via exposure of mice to the TLR2 agonist, PAM_3_CSK_4_, leads to an expansion of phenotypic bone marrow and spleen hematopoietic stem and progenitors (HSPCs), but a loss of bone marrow HSC function. Treatment of chimeric animals demonstrates that these effects are largely cell non-autonomous. Given the known role of G-CSF in mobilizing HSCs in response to TLR4 agonist,^8^ we assessed the contribution of G-CSF to the effects of PAM_3_CSK_4_ treatment on HSCs. Whereas PAM_3_CSK_4_ treatment is associated with increased serum G-CSF levels, loss of G-CSF signaling only partially mitigates the agonist-induced effects on HSCs. Further studies demonstrated that tumor necrosis factor-α (TNFα) significantly contributes to the TLR2 agonist-mediated cycling and expansion of spleen HSCs. Notably, inhibition of G-CSF in chimeric mice revealed a cell-autonomous role for TLR2 signaling in expanding phenotypic HSCs as well. Together, these data demonstrate that systemic exposure to a TLR2 ligand influences HSCs via both cell-autonomous and non-autonomous mechanisms, and reveal key differences between the effects of different TLR agonists on HSCs.

## Materials and methods

### Mice

C57BL/6J (CD45.2), C57BL/6 mice (B6.SJL-Ptprc* Pep3b BoyJ) carrying the CD45.1 allele, *Tlr2*^*−/−*^ (B6.129-Tlr2^tm1Kir^/J), *Ifnγ*^*−/−*^ (B6.129S7-Ifng^tm1Ts^/J), *Tnfα*^*−/−*^ (B6.129S-Tnf^tm1Fkl/J^), *Cxcr5*^*−/−*^ (B6.129S(Cg)-Cxcr5^tm1Lipp^/J) and *Ccl2*^*−/−*^ (B6.129S4-Ccl2^tm1Rol^/J) mice were obtained from the Jackson Laboratory (Bar Harbor, ME, USA). *Csf3r*^*−/−*^ mice were a gift of Dr Daniel Link (Washington University, St Louis, MO, USA). All mice were maintained on a C57BL/6J background. Sex- and age-matched mice were used in accordance with the guidelines of the Washington University Animal Studies Committee.

### TLR2 agonist and cytokine neutralizing antibody injections

PAM_3_CSK_4_ and PAM_2_CSK_4_ (Invivogen, San Diego, CA, USA) were reconstituted in sterile water and delivered by intraperitoneal injection. Unless otherwise indicated, mice were given 100 μg per dose, every 48 h × 3 doses and analyzed 24 h after the final dose. For experiments involving G-CSF and/or TNFα neutralization, 150 μg of G-CSF neutralizing antibody (clone 67604, R&D Systems, Minneapolis, MN, USA), 100 μg of TNFα neutralizing antibody (clone MP6-XT22, Biolegend, San Diego, CA, USA) or isotype control (clone 43414, R&D Systems) were injected intraperitoneally 90 min before each PAM_3_CSK_4_ injection.

### Flow cytometry for HSPC and chimerism analysis

Peripheral blood was obtained by retro–orbital venous plexus sampling. Bone marrow was isolated by centrifugation of leg bones at 3300 *g* for 1 min. Spleen cells were harvested by crushing through a 100 μM strainer. Cells were processed as previously described,^18^ and stained using the antibodies listed in Supplementary Materials. Cell counts were determined using a Countess automated cell counter (Invitrogen, Carlsbad, CA, USA). Stained cells were analyzed on a BD flow cytometer (BD, Franklin Lakes, NJ, USA) or the Gallios flow cytometer (Beckman Coulter, Indianapolis, IN, USA). Data were analyzed with FlowJo software (version 9.7; TreeStar, Ashland, OR, USA).

### Cell cycle analysis

For Ki-67 staining, bone marrow or spleen cells were stained for surface markers (see Supplementary Materials), fixed using the BD cytofix/cytoperm kit (BD), blocked with 5% goat serum, stained with mouse anti-human Ki-67 (clone B56; BD Pharmingen, San Diego, CA, USA), washed and resuspended in DAPI (4',6-diamidino-2-phenylindole)-containing FACS buffer. For analysis of 5-bromodeoxyuridine (BrdU) incorporation, mice were injected with BrdU (1 mg intraperitoneally q12h × 4 doses) starting 48 h before cell harvest. Cells were stained for surface markers, and then fixed and stained for anti-BrdU using the FITC BrdU flow kit (BD) according to the manufacturer's instructions.

### Long-term repopulating assays

For transplants involving sorted HSCs, bone marrow cells were obtained by crushing bones in phosphate-buffered saline with 0.1% bovine serum albumin. Spleens were gently crushed. Cells were filtered and stained using the panel of antibodies described in Supplementary Materials. c-Kit+ cells were enriched before sorting by selection with biotin-conjugated beads (Miltenyi Biotec, San Diego, CA, USA). Non-viable cells were excluded by DAPI staining. Twenty KSL SLAM donor HSCs were transplanted via retro–orbital injection, in addition to 3 00 000 recipient-type whole bone marrow support cells, into lethally irradiated recipient mice (2 doses of 550 cGy spaced 4 h apart). For whole bone marrow and whole spleen cell transplants, 1 × 10^6^ bone marrow cells or 2 × 10^6^ spleen cells were transplanted along with 1 × 10^6^ bone marrow competitor cells into irradiated recipients.

### Chimera generation

Chimeric mice were generated by transplanting a 1:1 mixture of whole bone marrow cells from wild-type (WT) and *Tlr2*^*−/−*^ donors into either WT or *Tlr2*^*−/−*^ recipients, as indicated. For Figure 4, WT or *Tlr2*^*−/−*^ bone marrow was transplanted alone into either WT or *Tlr2*^*−/−*^ recipients. A total of 2 × 10^6^ cells were injected retro–orbitally into lethally irradiated recipients. In all cases, cells were allowed to engraft for at least 11 weeks before treatment, and peripheral blood sampling of all chimeras was done before further analyses to ensure equal engraftment.

### Homing assay

2 × 10^6^ whole bone marrow cells were harvested from WT CD45.2 mice treated with PAM_3_CSK_4_ (100 μg per dose, every 48 h × 3 doses) or water alone and transplanted via retro–orbital injection into lethally irradiated WT CD45.1 mice. After 16 h, long bones were harvested (pelvis, femur, tibia and humerii), crushed, filtered and stained for flow cytometry.

### Cytokine measurements

Bone marrow was isolated by centrifugation of femurs and spleens were crushed through a 100 μm strainer. The total protein concentration was determined using the BCA Protein Assay (Pierce, Rockford, IL, USA). Blood was harvested by cardiac puncture, allowed to clot for 2 h, then spun for 10 min at 3300 *g*. Cytokine levels were determined using the Mouse Cytokine Array Panel A (R&D Systems) following the manufacturer's instructions. Membranes were exposed to X-ray film, and signal intensities were quantified with ImageJ (NIH, Bethesda, MD, USA). Serum G-CSF and TNFα levels were also measured using the mouse G-CSF quantikine enzyme-linked immunosorbent assay (ELISA) kit and the mouse TNFα quantikine ELISA kit, respectively (R&D Systems), following the manufacturer's instructions.

### Colony forming units in culture assay

A total of 50 000 spleen cells were plated in MethoCult GF M3434 (Stem Cell Technologies, Vancouver, BC, Canada). Colonies were scored after 7 days of growth at 37 °C in a humidified chamber with 5% CO_2_.

### Statistical analysis

Data are presented as mean±s.e.m., unless otherwise stated. Statistical significance was assessed using an unpaired, two-tailed Student's *t-*test or two-sided analysis of variance. Repopulating unit calculations were determined using the equation: RU=([%chimerism of test donor-derived cells] × [no. of competitor cells] × 10^*−*5^)/%chimerism of competitor-derived cells.^19^

## Results

### TLR2 agonist treatment leads to expansion of bone marrow phenotypic HSCs but loss of BM repopulating activity

**Figure 1:**
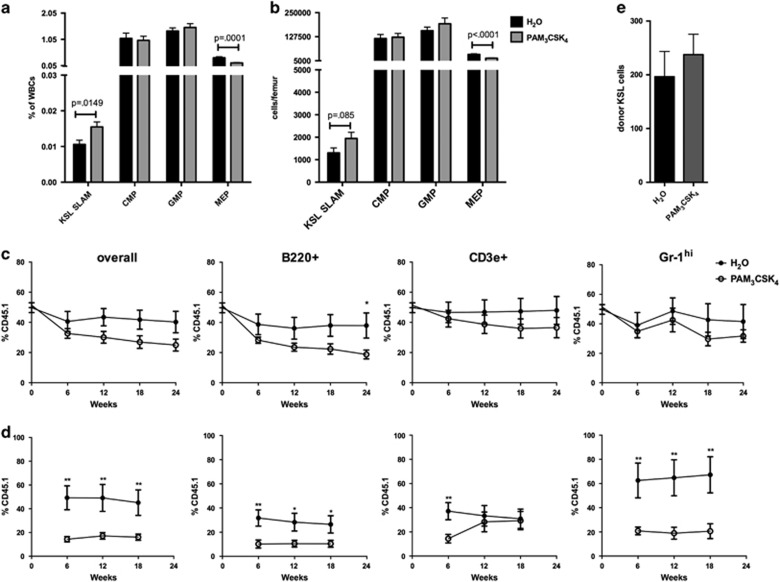
TLR2 agonist treatment leads to increased bone marrow phenotypic HSC frequency but loss of bone marrow repopulating activity. WT mice (6–8 weeks old) were treated with PAM_3_CSK_4_ (100 μg intraperitoneally (i.p.) q48 h × 3 doses, analyzed 24 h after final dose). Shown are the frequency (**a**) and absolute numbers (**b**) of bone marrow HSPCs as determined by flow cytometry using the gating strategy outlined in Supplementary Figure 1 (*n*= 12–13 mice per treatment group). (**c**) Whole bone marrow from PAM_3_CSK_4_-treated mice or water-treated controls (CD45.1) was transplanted in a 1:1 ratio with untreated competitor bone marrow (CD45.2) into lethally irradiated recipients (CD45.1/CD45.2). Shown are the frequency of donor cells (CD45.1+ single positive), including overall leukocytes, B cells (B220+), T cells (CD3e+) and neutrophils (Gr-1 hi), in the peripheral blood of primary recipients over time. (**d**) At 24 weeks post transplant, bone marrow from primary recipients was pooled and transplanted into secondary recipients. Shown is the frequency of donor cells (CD45.1+ single positive) in the peripheral blood of these secondary recipients over time. Data represent 8–12 recipient mice per treatment group from 2 independent experiments. *P<0.05; **P<0.01. (**e**) To assess homing, 2 × 10^6^ whole bone marrow cells from PAM_3_CSK_4_-treated mice or water controls were transplanted into lethally irradiated recipients. Shown are the total number of KSL cells detected from the long bones (femurs, tibias, humerii) and pelvis 16 h later (*n*= 5 mice per group). For all panels, error bars represent mean±s.e.m., and *P-*values were determined by the two-tailed Student's *t*-test.

TLR2 functions as a heterodimer with TLR1 or TLR6.^20^ To determine the effects of systemic TLR2 agonist exposure on HSCs, WT mice were treated with the TLR1/2 agonist PAM_3_CSK_4_ (3 doses of 100 μg intraperitoneally every other day). Bone marrow and spleen cells were harvested 24 h following the final dose and analyzed by flow cytometry (Supplementary Figure 1). We observed a modest increase in the frequency of bone marrow HSCs (c-Kit+ Sca-1+ Lineage− CD150+ CD48− cells, ‘KSL SLAM' or c-Kit+ Lineage− CD150+ CD48−) and a decrease in megakaryocyte–erythroid progenitors (c-Kit+ Sca-1− Lineage− CD34− CD16/32− Figures 1a and b and Supplementary Figure 1) in agonist-treated mice compared with controls. To determine HSC function, competitive repopulating assays were performed in which whole bone marrow from PAM_3_CSK_4_- or water control-treated mice was transplanted in a 1:1 ratio with untreated competitor marrow into lethally irradiated recipients. The bone marrow from PAM_3_CSK_4_-treated mice showed impairment in B220+ (B cell) engraftment in primary recipients (Figure 1c). To test HSC self-renewal, bone marrow from the primary recipients was transplanted into secondary recipient mice. Peripheral blood sampling of the secondary recipients showed a significant reduction in test donor chimerism from the PAM_3_CSK_4_-treated group versus controls (Figure 1d), demonstrating reduced HSC self-renewal. Finally, to assess HSPC homing, 2 × 10^6^ whole bone marrow cells from PAM_3_CSK_4_-treated WT (CD45.2) mice or water-treated controls were injected into lethally irradiated CD45.1 recipients and KSL cells were enumerated 16 h later (Figure 1e). We found no difference in the number of homed donor cells between the groups, suggesting that the loss of HSC function upon PAM_3_CSK_4_ treatment cannot be explained by lack of HSPC homing.

### TLR2 signaling leads to expansion of spleen HSPCs

**Figure 2:**
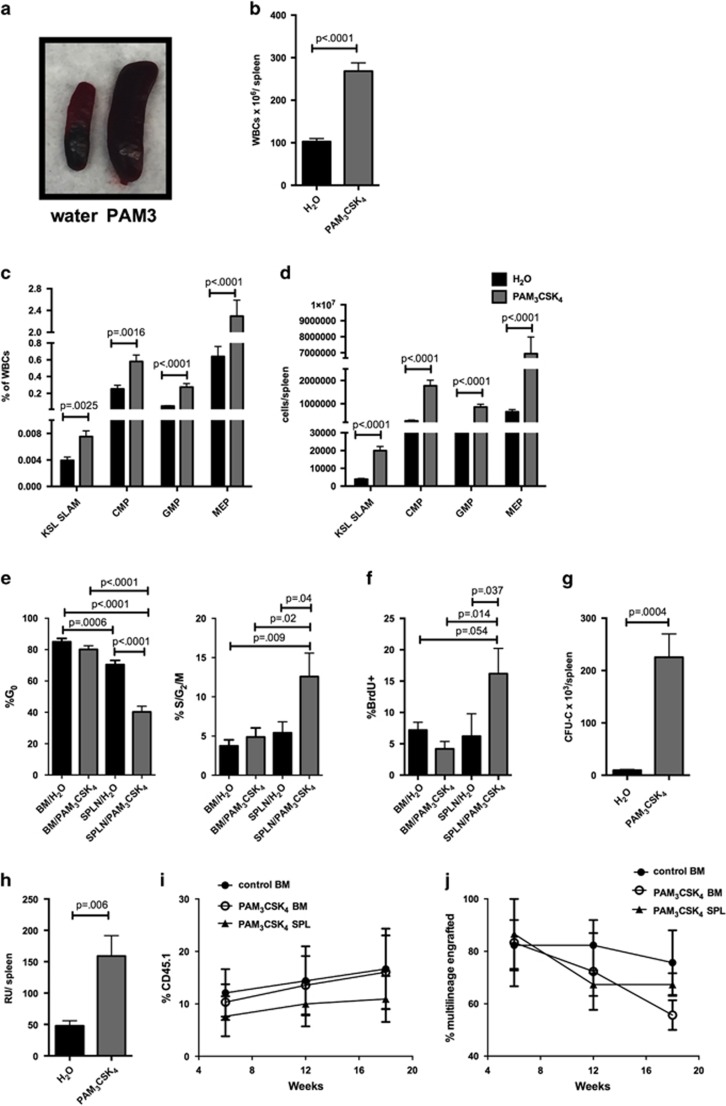
TLR2 agonist treatment leads to marked expansion of spleen HSPCs. WT mice (6–8 weeks old) were treated with PAM_3_CSK_4_ (100 μg intraperitoneally (i.p.) q48 h × 3 doses, analyzed 24 h after final dose). (**a**) Representative spleens from control water-treated (left) and PAM_3_CSK_4_-treated (right) mice. Shown are the total leukocytes per spleen (**b**) and the frequency (**c**) and absolute numbers (**d**) of KSL SLAM cells, common myeloid progenitors (CMPs), granulocyte–monocyte progenitors (GMPs) and megakaryocyte–erythroid progenitors (MEPs) in the spleen as determined by flow cytometry (*n*=11–13 mice per treatment group). The cell cycle status of KSL SLAM cells in the bone marrow and spleen was determined by flow cytometry using Ki-67 and DAPI (**e**) or BrdU incorporation (**f**) (*n*=7–10 mice per group). (**g**) Whole spleen cells from PAM_3_CSK_4_-treated mice or water-treated controls were plated on complete methylcellulose, and colonies counted after 7 days of growth at 37 °C (*n*=7 mice per group). (**h**) To determine HSC function, 2 × 10^6^ whole spleen cells (CD45.1) from control or PAM_3_CSK_4_-treated mice were transplanted along with 1 × 10^6^ whole bone marrow competitor cells (CD45.2) into lethally irradiated WT recipients (CD45.1/CD45.2). Shown are total repopulating units per spleen at 18 weeks post transplant. Repopulating units were determined using the equation RU=([% chimerism of test donor-derived cells] × [no. of competitor cells] × 10^*−*5^)/% chimerism of competitor-derived cells. The result was then multiplied by total spleen WBCs to obtain RUs/spleen. Data represent seven mice per treatment group from two independent experiments. (**i**, **j**) Twenty KSL SLAM HSCs (CD45.1) from the spleens or bone marrow of PAM_3_CSK_4_-treated mice were transplanted with 3 × 10^5^ whole marrow support cells (CD45.2) into irradiated recipients. Shown are % donor (CD45.1) leukocytes in the recipients (**i**) and % of recipients with multilineage (⩾1% in myeloid and lymphoid lineages) donor (CD45.1) engraftment (**j**). Data represent 11–12 mice per group from 3 independent experiments. For all panels, error bars represent mean±s.e.m.. Data in (**i** and **j**) were analyzed by two-way analysis of variance (ANOVA) with multiple comparisons, and all other *P-*values were determined by two-tailed Student's *t*-test.

The spleens of PAM_3_CSK_4_-treated mice were significantly enlarged (Figures 2a and b) and had a marked increase in HSPC populations (Figures 2c and d and Supplementary Figure 1). Cell cycle analysis of spleen HSCs using either Ki-67 and DAPI or BrdU incorporation demonstrated that, in contrast to the bone marrow, the KSL SLAM cells in the spleen were significantly less quiescent upon PAM_3_CSK_4_ treatment (Figures 2e and f and Supplementary Figure 2), suggesting that local proliferation (as opposed to mobilization alone) may contribute to this expansion. Consistent with the increased myeloid progenitors seen by flow cytometry, the spleens of PAM_3_CSK_4_-treated mice displayed a marked increase in progenitor colony formation in methylcellulose (Figure 2g). Treatment with lower doses of PAM_3_CSK_4_ demonstrated that this expansion of HSPCs is dose dependent (Supplementary Figure 3). Treatment with the specific TLR2/6 agonist PAM_2_CSK_4_ produced similar results, with expansion of bone marrow and spleen HSPCs (Supplementary Figure 4). Finally, to determine whether TLR2 agonist exposure increases functional HSCs in the spleen, we transplanted either whole spleen cells or sorted HSCs into lethally irradiated recipient mice. Transplantation of whole spleen cells revealed a significant increase in total repopulating units per spleen in agonist-treated mice compared with controls (Figure 2h). Transplantation of sorted KSL SLAM cells (20 HSCs (CD45.1) with 3 × 10^5^ whole marrow support cells (CD45.2) into irradiated recipients) from the spleens or bone marrow of PAM_3_CSK_4_-treated mice demonstrated that the spleen KSL SLAM cells have similar function to bone marrow KSL SLAM cells in PAM_3_CSK_4_-treated mice (Figures 2I and j). Thus, functional HSCs increase in the spleen upon TLR2 agonist exposure.

### TLR2 agonist effects on HSC expansion and function are, at least in part, cell non-autonomous

**Figure 3:**
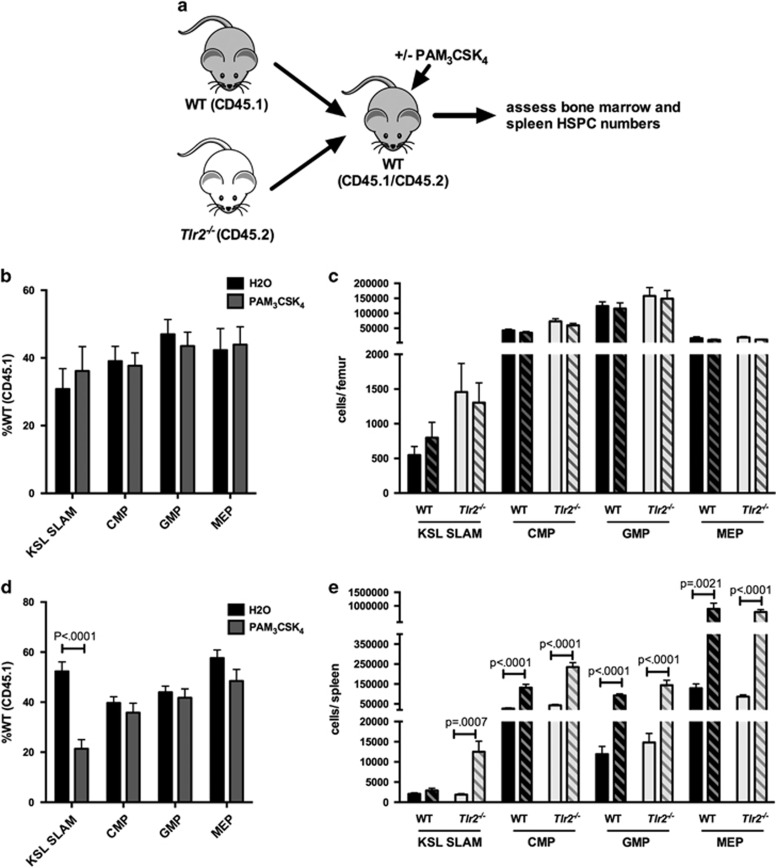
TLR2 agonist effects on HSPCs are, at least in part, cell non-autonomous. (**a**) Chimeric animals were generated by transplanting equal numbers of WT (CD45.1) and *Tlr2*^*−/−*^ (CD45.2) bone marrow cells into lethally irradiated WT (CD45.1/CD45.2) recipients. After allowing time for engraftment (12 weeks), recipients were treated with PAM_3_CSK_4_ (100 μg intraperitoneally (i.p.) q48 h × 3 doses) and the relative frequencies (**b**, **d**) and absolute numbers (**c**, **e**) of HSPCs in the bone marrow (**b**, **c**) and spleen (**d**, **e**) were determined by flow cytometry. (**b**) The frequency of WT (CD45.1) HSPCs for each indicated cell type in the bone marrow after treatment is shown. (**c**) Absolute numbers of cells per femur after treatment of chimeras is shown. Dark bars indicate WT (CD45.1) cells, and light bars indicate *Tlr2*^*−/−*^ (CD45.2) cells. Solid bars indicate water-treated controls, and hatched bars indicate PAM_3_CSK_4_-treated mice (*n*=10 mice per treatment group). (**d**) The frequency of WT (CD45.1) HSPCs for each indicated cell type in the spleen after treatment is shown. (**e**) Absolute numbers of cells per spleen after treatment of chimeras is shown. Dark bars indicate WT (CD45.1) cells, and light bars indicate *Tlr2*^*−/−*^ (CD45.2) cells. Solid bars indicate water-treated controls, and hatched bars indicate PAM_3_CSK_4_-treated mice (*n*=10 mice per treatment group). For all panels, error bars represent mean±s.e.m., and *P-*values were determined by the two-tailed Student's *t*-test.

As TLR2 is expressed on multiple hematopoietic and stromal cell populations in addition to HSCs, the effects of systemic TLR2 agonist treatment could be either cell autonomous or cell non-autonomous. A recent study demonstrated that exposure of mice to a TLR4 ligand mobilizes HSCs in a cell-non-autonomous, G-CSF-dependent manner.^8^ To determine whether the effects of TLR2 signaling are similarly non-autonomous, we generated chimeric mice in which a 1:1 mixture of *Tlr2*^*−/−*^ (CD45.2) and WT (CD45.1) bone marrow cells were transplanted into WT recipients (Figure 3a). Chimeras were treated as above with PAM_3_CSK_4_, and HSPCs were analyzed by flow cytometry. In addition, bone marrow from these mice was transplanted into new irradiated recipients to assess HSC function. We reasoned that if TLR2 signaling regulates HSCs in a cell-autonomous manner, we would observe a preferential effect of agonist treatment on WT cells, whereas if the effects of TLR2 ligation were non-autonomous, we would find a similar effect of treatment on both *Tlr2*^*−/−*^ and WT HSCs. Although there was not a significant increase in bone marrow HSCs from either population in these chimeras (Figures 3b and c), PAM_3_CSK_4_ treatment resulted in a marked increase in both WT and *Tlr2*^*−/−*^ HSPCs in the spleen (Figures 3d and e), suggesting that the spleen expansion is mediated by extrinsic factors. In fact, we actually saw a preferential mobilization of *Tlr2*^*−/−*^ HSCs in our chimeras. The reason for this is unclear, however a similar phenomenon was reported for *Tlr4*^*−/−*^ HSCs mobilized by *Escherichia coli*,^8^ and we previously found that *Tlr2*^*−/−*^ HSCs mobilize more robustly than WT cells upon G-CSF exposure, suggesting that they may be more responsive to mobilizing stimuli.^16^

To assess whether the loss of bone marrow repopulating activity is cell autonomous versus non-autonomous, we transplanted bone marrow from the treated chimeras into new irradiated recipients. Again, we reasoned that if the effects of TLR2 signaling on HSC function are cell autonomous, we would observe a preferential defect in the WT cells (that is, a decrease in %CD45.1 upon PAM_3_CSK_4_ treatment), whereas if the effects of TLR2 ligation are non-autonomous, we would find a similar effect of treatment on both *Tlr2*^*−/−*^ and WT HSCs (that is, no difference in chimerism). Analysis of the peripheral blood chimerism of these secondary recipients showed no differences between treatment groups (Supplementary Figure 5), suggesting that the functional deficit described in Figure 1 is influenced by non-autonomous TLR2 signaling. Thus, as the effects of PAM_3_CSK_4_ on HSPCs are observed in cells lacking TLR2 as well as WT cells, these effects must be, at least in part, cell non-autonomous.

### Spleen HSPC expansion upon TLR2 agonist exposure is mediated by non-autonomous effects of TLR2 signaling in both radioresistant and radiosensitive cell populations

**Figure 4:**
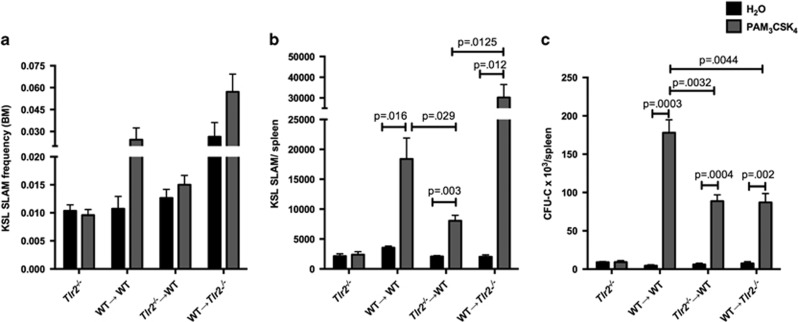
The effects of TLR2 agonist exposure are mediated by non-autonomous TLR2 signaling in both radioresistant and radiosensitive populations. Chimeric mice were generated by transplanting WT bone marrow into WT recipients (WT→WT), *Tlr2*^*−/−*^ bone marrow into WT recipients (*Tlr2*^*−/−*^→WT) and WT bone marrow into *Tlr2*^*−/−*^ recipients (WT→*Tlr2*^*−/−*^). After allowing time for engraftment (11 weeks), mice were treated with PAM_3_CSK_4_ (100 μg intraperitoneally (i.p.) q48 h × 3 doses). Shown are KSL SLAM frequency in the bone marrow (**a**) and KSL SLAM cells per spleen (**b**), as determined by flow cytometry, in chimeric mice and in constitutional *Tlr2*^*−/−*^ controls. (**c**) Spleen cells from chimeric mice and constitutional *Tlr2*^*−/−*^ controls were plated in complete methylcellulose media and myeloid colony formation was scored after 7 days of growth at 37 °C (*n*=3–4 mice per group). For all panels, error bars represent mean±s.e.m., and *P-*values were determined by the two-tailed Student's *t*-test.

We next generated chimeras to determine whether stromal versus hematopoietic TLR2 signaling mediates HSPC expansion. Specifically, we transplanted WT bone marrow into *Tlr2*^*−/−*^ recipients (WT→*Tlr2*^*−/−*^), *Tlr2*^*−/−*^ marrow into WT recipients (*Tlr2*^*−/−*^→WT) or WT into WT (WT→WT). We treated the mice with PAM_3_CSK_4_ as before and assessed bone marrow and spleen HSPCs by flow cytometry. We observed a nonsignificant trend toward increased HSC frequency only in cases where hematopoietic cells expressed TLR2 (WT→WT and WT→*Tlr2*^*−/−*^; Figure 4a); however, spleen HSPCs increased in all three chimeric groups (Figures 4b and c), suggesting that TLR2 expression on either radioresistant (that is, mostly stromal) or radiosensitive (hematopoietic) cells is sufficient to induce some mobilization (or expansion) of spleen HSPCs.

### TLR2 agonist treatment leads to G-CSF production by both radioresistant and radiosensitive cell populations

**Figure 5:**
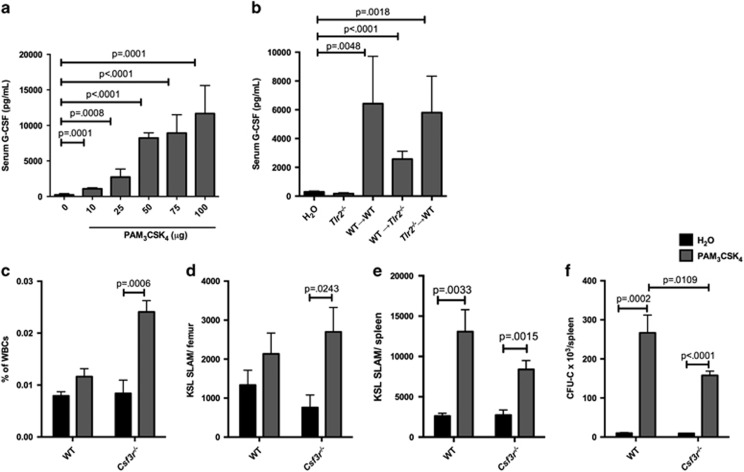
G-CSF production partially accounts for the effects of TLR2 agonist exposure on HSPCs. (**a**) WT mice (6–8 weeks old) were given a single intraperitoneal (i.p.) injection of PAM_3_CSK_4_ at the indicated doses, and serum G-CSF levels were determined by ELISA 12 h later (*n*=4–6 mice per treatment level). (**b**) Chimeric mice as described in Figure 4 were given a single i.p. injection of 100 μg PAM_3_CSK_4_, and serum G-CSF levels were determined by ELISA (*n*=3 mice per group, with the exception of the water group (*n*=9) that represents pooled controls from all of the treatment groups. (**c**) WT or *Csf3r*^*−/−*^ mice were treated with PAM_3_CSK_4_ (100 μg i.p. q48 h × 3 doses, analyzed 24 h after final dose). Shown are the frequency (**c**) and absolute numbers (**d**) of KSL SLAM cells in the bone marrow. Also shown are KSL SLAM cells per spleen (**e**). (**f**) Whole spleen cells from PAM_3_CSK_4_-treated mice or water-treated controls were plated on complete methylcellulose, and colonies counted after 7 days of growth at 37 °C (*n*=6–7 mice per group). For all panels, error bars represent mean±s.e.m., and *P-*values were determined by the two-tailed Student's *t*-test.

To identify potential mediators of the cell-non-autonomous effects of TLR2 signaling on HSPCs, we performed cytokine arrays on WT mouse serum, bone marrow and spleen after systemic treatment with PAM_3_CSK_4_. Serum levels of G-CSF, CXCL13, IP-10/CXCL10 and monocyte chemoattractant protein (MCP-1/CCL2) were the most markedly increased in treated mice compared with controls (Supplementary Figure 6). As noted above, recent reports have demonstrated that the TLR4 ligand lipopolysaccharide induces G-CSF production from endothelial cells, and G-CSF mediates the mobilization of HSCs in response to lipopolysaccharide.^8, 9^ We therefore first considered that G-CSF may similarly mediate HSC mobilization in response to TLR2 agonist. Indeed, PAM_3_CSK_4_ exposure is associated with a dose-dependent increase in serum G-CSF (Figure 5a). Furthermore, we observed an increase in serum G-CSF in all of our chimeras (WT→WT, WT→*Tlr2*^*−/−*^ and *Tlr2*^*−/−*^→WT), but not in parent *Tlr2*^*−/−*^ mice (Figure 5b), suggesting that both radioresistant and radiosensitive cell populations serve as sources of this cytokine.

### Suppression of G-CSF signaling partially mitigates the effects of TLR2 agonist exposure on HSPCs

To assess the contribution of G-CSF signaling to the effects of TLR2 agonist treatment on HSCs, we treated mice lacking the G-CSF receptor (*Csf3r*^*−/−*^ mice) with PAM_3_CSK_4_. Although bone marrow HSCs expanded in response to PAM_3_CSK_4_ in the absence of G-CSF signaling (Figures 5c and d), loss of this signaling leads to a modest reduction in spleen myeloid progenitor cell expansion, specifically resulting in reduced expansion of spleen common myeloid progenitors, granulocyte–monocyte progenitors and myeloid progenitor colony-forming units in response to PAM_3_CSK_4_ compared with WT (Figure 5f and Supplementary Figure 7). A similar reduction in PAM_3_CSK_4_-mediated spleen myeloid progenitor expansion was observed when WT mice were treated with a G-CSF neutralizing antibody (Supplementary Figure 7). Of note, we still observed a significant increase in spleen HSCs and progenitors in the absence of G-CSF signaling (Figure 5e and Supplementary Figure 7), suggesting that G-CSF is not the sole mediator of this increase. In fact, transplantation of whole spleen cells from mice treated with PAM_3_CSK_4_ and G-CSF neutralizing antibody revealed no loss of total spleen repopulating units (Supplementary Figure 7).

### Cell-autonomous TLR2 signaling contributes to spleen HSC expansion

**Figure 6:**
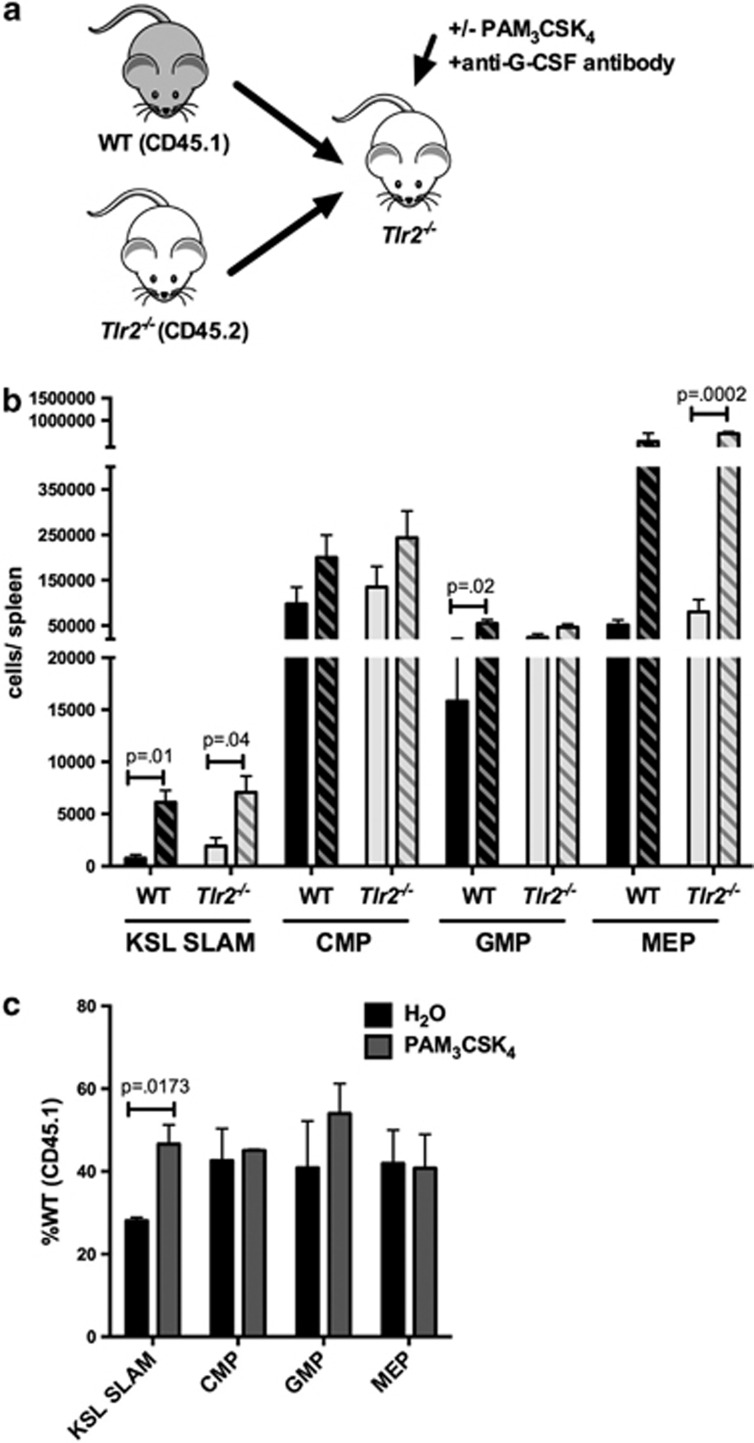
Cell-autonomous TLR2 signaling contributes to PAM_3_CSK_4_-mediated HSC expansion. (**a**) Chimeric animals were generated by transplanting equal numbers of WT (CD45.1) and *Tlr2*^*−/−*^ (CD45.2) bone marrow cells into lethally irradiated *Tlr2*^*−/−*^ (CD45.2) recipients. (**b**) After allowing time for engraftment (12 weeks), recipients were treated with PAM_3_CSK_4_ (100 μg intraperitoneally (i.p.) q48 h × 3 doses) and a G-CSF neutralizing antibody and the absolute numbers of KSL SLAM, common myeloid progenitor (CMP), granulocyte–monocyte progenitor (GMP) and megakaryocyte–erythroid progenitor (MEP) cells per spleen for each donor cell type were determined by flow cytometry. Shown are cells/spleen for the indicated populations. Dark bars indicate WT (CD45.1) cells, and light bars indicate *Tlr2*^*−/−*^ (CD45.2) cells. Solid bars indicate water-treated controls, and hatched bars indicate PAM_3_CSK_4_-treated mice. (**c**) The relative frequencies of wild-type donor (CD45.1) HSPCs are shown for PAM_3_CSK_4_-treated or water control-treated chimeric mice. Data represent three chimeras from each treatment group from three separate experiments, and *P-*values were determined by two-tailed Student's *t*-test. Error bars represent mean±s.e.m.

As G-CSF only partially accounts for the expansion of spleen HSPCs in response to PAM_3_CSK_4_, other extrinsic factors, or cell-autonomous TLR2 signaling, must account for the remaining increase. To address whether there is a cell-autonomous component to this expansion, we treated chimeric mice (WT+*Tlr2*^*−/−*^ bone marrow transplanted into *Tlr2*^*−/−*^ recipients, Figure 6a) with PAM_3_CSK_4_ and a G-CSF neutralizing antibody, thus minimizing the effects of G-CSF. Again, we see expansion of both WT and *Tlr2*^*−/−*^ HSPCs in the spleen (Figure 6b), supporting a role for G-CSF-independent, hematopoietic-mediated non-autonomous TLR2 signaling in this process. However, we observed a relatively greater expansion of WT HSCs compared with *Tlr2*^*−/−*^ HSCs (Figure 6c), suggesting that cell-autonomous TLR2 signaling contributes to HSC expansion. Thus, although PAM_3_CSK_4_ clearly affects cells lacking TLR2 (reflecting indirect, or cell-non-autonomous effects), the more robust expansion of WT cells compared with *Tlr2*^*−/−*^ cells in this experiment demonstrates that cell-autonomous signaling also contributes to this effect of PAM_3_CSK_4_ on HSCs.

### TNFα contributes to the TLR2 agonist-mediated expansion of HSPCs in the spleen

**Figure 7:**
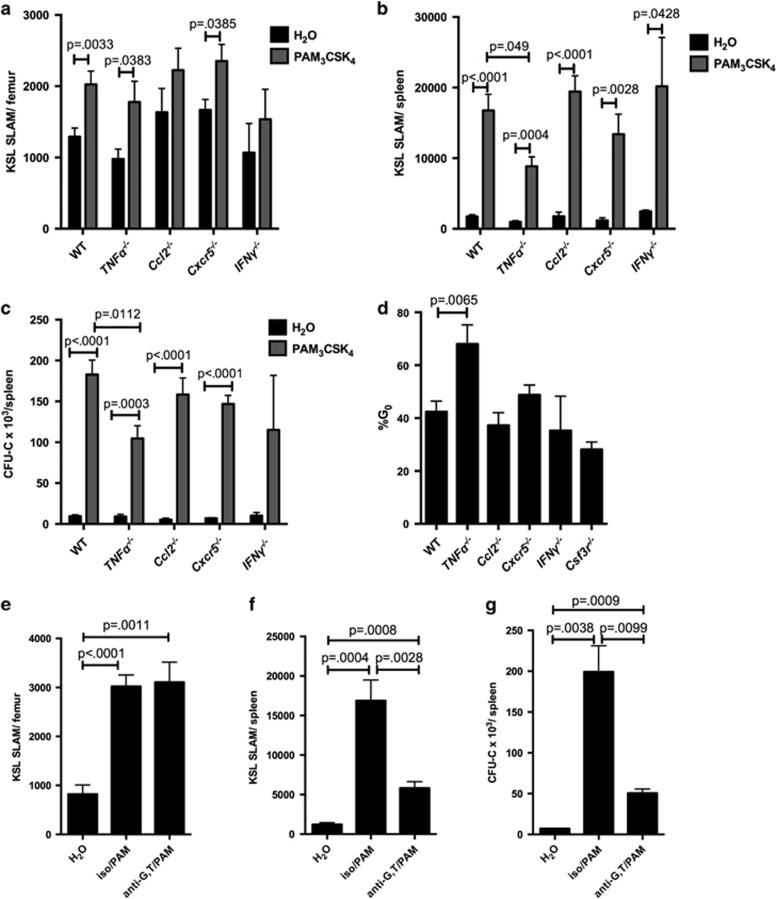
TNFα contributes to PAM_3_CSK_4_-mediated spleen HSPC expansion. WT, *TNFα*^*−*/*−*^, *Ccl2*^*−*/*−*^, *Cxcr5*^*−*/*−*^ and *IFNγ*^*−*/*−*^ mice (6–8 weeks old) were treated with PAM_3_CSK_4_ (100 μg intraperitoneally (i.p.) q48 h × 3 doses, analyzed 24 h after final dose) or water alone. Shown are the KSL SLAM cells per femur (**a**) and spleen (**b**). (**c**) Whole spleen cells from PAM_3_CSK_4_-treated mice or water-treated controls were plated on complete methylcellulose, and colonies counted after 7 days of growth at 37 °C. (**d**) Following PAM_3_CSK_4_ treatment, the cell cycle status of KSL SLAM cells in the spleen of mice of the indicated genotypes were determined by flow cytometry using Ki-67 and DAPI. (*n*=4–12 mice per group). (**e–g**) WT mice were treated with PAM_3_CSK_4_ as described above, and in addition some mice received G-CSF and TNFα neutralizing antibodies or isotype control antibody before PAM_3_CSK_4_ injections. KSL SLAM cells in the bone marrow (**e**) and spleen (**f**) were determined by flow cytometry. In addition, whole spleen cells were plated on complete methylcellulose, and colonies counted after 7 days of growth at 37 °C (**g**); *n*=5–6 mice per group. For all panels, error bars represent mean±s.e.m., and *P-*values were determined by the two-tailed Student's *t*-test.

We next considered whether the other cytokines elevated upon PAM_3_CSK_4_ treatment contribute to the remaining non-autonomous effects of TLR2 signaling on HSCs (Supplementary Figure 6). To this end, we assessed the response of *Ccl2*^*−*/*−*^, *Cxcr5*^*−*/*−*^ (CXCR5 is the receptor for CXCL13), *IFNγ*^*−*/*−*^ and *TNFα*^*−*/*−*^ mice to PAM_3_CSK_4_ exposure. We assessed a small number of *Cxcl10*^*−/−*^ mice as well with no obvious difference in TLR2- mediated effects from WT (data not shown). As CXCL10 is regulated by interferon-γ (IFNγ) and TNFα, and these cytokines can regulate HSCs,^21, 22, 23^ we tested their contribution as well (in fact, serum TNFα levels increased by ELISA upon PAM_3_CSK_4_ treatment; Supplementary Figure 8). Notably, loss of TNFα results in a significant decrease in the magnitude of the PAM_3_CSK_4_- mediated expansion of spleen HSCs and progenitors compared with WT (Figures 7b and c and Supplementary Figure 8), and spleen HSC cycling in response to the TLR2 agonist is mitigated in the absence of TNFα (Figure 7d). Furthermore, treatment of mice with neutralizing antibodies to both G-CSF and TNFα before PAM_3_CSK_4_ injection reduced the expansion of HSCs in the spleen by nearly threefold, and markedly decreased spleen common myeloid progenitors, granulocyte–monocyte progenitors and progenitor colony-forming units (Figures 7e–g and Supplementary Figure 8), further implicating these cytokines as regulators of spleen HSPC mobilization/expansion in response to TLR2 ligand.

## Discussion

Although multiple TLRs are expressed on HSCs, most notably TLR2 and TLR4,^10^ most of the published studies have focused on the effects of TLR4 stimulation on HSCs. We recently reported that TLR2 is not necessary to maintain normal HSC function,^16^ and herein we sought to determine the effects of systemic TLR2 ligand exposure on HSCs. Our data demonstrate that indeed TLR2 stimulation affects HSC expansion and function, and suggest that these effects are mediated by both cell-autonomous and cell-non-autonomous mechanisms.

Recent studies have shown that exposure of mice to systemic TLR4 ligands leads to HSC expansion and mobilization, and a loss of bone marrow HSC function.^4, 5, 8, 24, 25^ Mechanistic studies indicate that TLR4 stimulation leads to G-CSF production from endothelial cells,^9^ and G-CSF signaling blockade abrogates TLR4-induced HSC accumulation in the spleen.^8^ Like TLR4, stimulation of TLR2 leads to HSPC expansion, loss of HSC function and G-CSF production. However, in response to TLR2 agonist, G-CSF is produced from both radioresistant and radiosensitive populations, suggesting it is not primarily endothelial in origin. In addition, suppression of G-CSF signaling only partly mitigates the expansion of myeloid progenitors in the spleen and does not significantly affect HSCs. Further studies demonstrated that TNFα contributes to the cell-non-autonomous effects of TLR2 agonist exposure on HSPCs. The identity of the cell types that mediate these non-autonomous effects await further studies using mice with conditional deletion of TLR2.

Our chimeric animal studies with G-CSF inhibition also support a role for cell-autonomous TLR2 signaling in the expansion of HSCs. Megias *et al.*^26^ previously demonstrated that purified WT c-Kit+ Sca-1+ Lineage− HSPCs transferred into *Tlr2*^*−/−*^-deficient recipients differentiate into macrophages in response to short-term treatment with PAM_3_CSK_4_, suggesting that HSPCs can directly responding to TLR signals *in vivo*. Joo *et al.*^27^ similarly demonstrated that WT c-Kit+ Lineage− HSPCs transplanted into *Tlr2*^*−/−*^-deficient mice differentiated into myeloid lineage cells in response to TLR2 agonist. Together with prior *in vitro* studies demonstrating that TLR2 stimulation directs myeloid differentiation of HSPCs,^1, 3^ our data reinforce the idea that TLR2 agonists have cell-autonomous effects on HSCs. These data highlight an important point that the effects of TLR signals on HSCs may differ depending on the particular agonist/TLR, with TLR4 signals exerting largely G-CSF-dependent, non-cell-autonomous effects on HSCs and TLR2 signals exerting primarily TNFα-dependent effects, with a more minor contribution from G-CSF and cell-autonomous effects.

Interestingly, we find that, unlike bone marrow HSCs, spleen HSCs are significantly less quiescent upon TLR2 ligand exposure. Thus, in contrast to the primarily mobilization-mediated increase in spleen HSCs upon TLR4 ligation,^8^ TLR2 signaling appears to stimulate local proliferation of spleen HSCs. This TLR2 agonist-induced spleen HSC cycling is lost in *TNFα*^*−*/*−*^ mice, implicating TNFα in this process. The biologic significance of this local proliferation and the reason for the differential effects of TLR2 agonist exposure on the cycling of bone marrow versus spleen HSCs is not known. Of note, Granick *et al.*^28^ recently reported that proliferation of HSPCs in skin wounds in response to the TLR2 agonist *Staphylococcus* *aureus* contributes significantly to the production of neutrophils and resolution of local infection, supporting a role for TLR2 signaling in the regulation of extramedullary hematopoiesis.

The accumulating data on TLR agonist effects on HSCs suggest that TLR signals may, in the short term, influence HSC proliferation and differentiation to optimize the immune response to an acute insult. In theory, enhanced cycling, mobilization and differentiation of HSCs during acute infection could help to ensure that the proper cadre of effector cells are produced to eliminate the offending stimulus. Chronic or aberrant exposure to these signals, however, may lead to dysfunctional hematopoiesis and contribute to bone marrow failure or malignancy. Enhanced TLR2 signaling has recently been implicated in MDS.^15^ Our data support a role for TLR2 signaling in the regulation of HSCs, with agonist exposure leading to expansion of HSCs in the bone marrow and spleen, but a reduction in HSC self-renewal. These findings suggest the possibility that enhanced TLR2 signaling may contribute to the ineffective hematopoiesis characteristic of MDS. The role of TLR2 signaling in this disease and the contribution of TLR2 signaling from different cell types to the effects of TLR2 agonist exposure on normal and premalignant HSCs awaits further study.
