# Supplementary material for: Systemic TLR2 agonist exposure regulates hematopoietic stem cells via cell-autonomous and cell-non-autonomous mechanisms
Source: Blood Cancer J. 2016 Jun 17;6(6):e437–. doi: 10.1038/bcj.2016.45 (PMC5141360; doi:10.1038/bcj.2016.45)
Supplement: Supplementary Information [file bcj201645x1.doc]

SUPPLEMENTARY MATERIALS

Antibodies used in this study:

The following antibodies were used to label HSPCs for analysis (all antibodies were from eBiosciences, except where indicated): anti–mouse Ly6A/E (Sca-1) PerCP-Cy5.5 (clone D7), anti–mouse CD117 (c-Kit) APC–eFluor 780 (clone 2B8), anti–mouse CD34 FITC (clone RAM34), anti–mouse CD16/32 eFluor 450 (clone 93), anti–mouse Flt3 (Flk-2) APC (clone A2F10), anti-mouse CD48 PE-Cy7 (clone HM48-1), anti-mouse CD150 PE (clone TC15-12F12.2, Biolegend, San Diego, CA), anti–mouse/human CD45R (B220) biotin (clone RA3-6B2), anti–mouse CD3e biotin (clone 145-2C11), anti–mouse Ly-6G (Gr-1) biotin (clone RB6–8C5), anti–mouse Ter119 biotin (clone TER-119), and streptavidin eFluor 605NC.

For peripheral blood chimerism analyses, the following antibodies were used: anti–mouse CD45.1 (clone A20), anti–mouse CD45.2 (clone 104), anti–mouse/human CD45R (B220; clone RA3-6B2), anti–mouse CD3e (clone 145-2C11), and anti–mouse Ly-6G (Gr-1; clone RB6-8C5).

The following antibodies were used to label KSL SLAM cells prior to sorting for experiments involving transplantation of sorted HSCs (all antibodies were from eBioscience, San Diego, CA, USA, unless indicated otherwise): anti-mouse CD150 PE (clone TC15-12F12.2, Biolegend, San Diego, CA, USA), anti-mouse cKit (CD117) Biotin (clone 2B8), anti-mouse APC CD48 (clone HM48-1), anti-mouse Ly6A/E (Sca-1) PerCP-Cy5.5 (clone D7), anti-mouse/human CD45R (B220) FITC (clone RA3-6B2), anti-mouse Ly6G (Gr-1) FITC (clone RB6-8C5), anti-mouse TER-119 FITC, (clone TER-119), anti-mouse CD3e FITC (clone 145-2C11), and Streptavidin PE-Cy7.


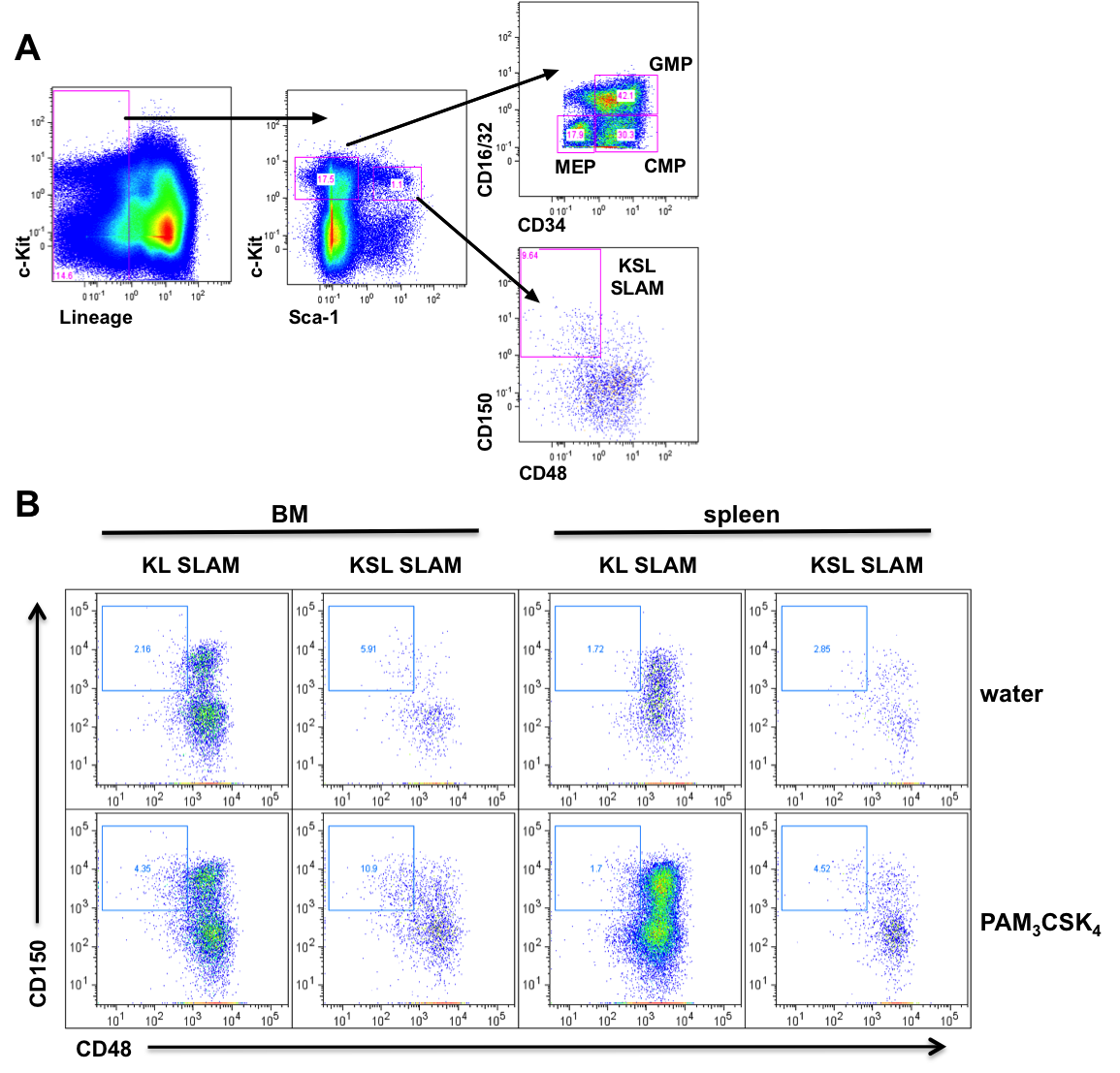


**Supplementary Figure 1**. **HSCs expand upon systemic exposure to TLR2 agonist.** (**A**) Shown are representative flow cytometry plots from mouse bone marrow demonstrating the gates applied to identify HSPCs throughout the study. (**B**) Shown are representative plots from the bone marrow and spleen of a PAM_3_CSK_4_ treated WT mouse (100 µg/dose x 3 doses IP spaced 48 hours apart) versus a water treated control. Populations are previously gated for Lineage-, and either further gated on c-Kit+ Sca-1+ (for KSL SLAM) or just c-Kit+ alone (for KL SLAM).


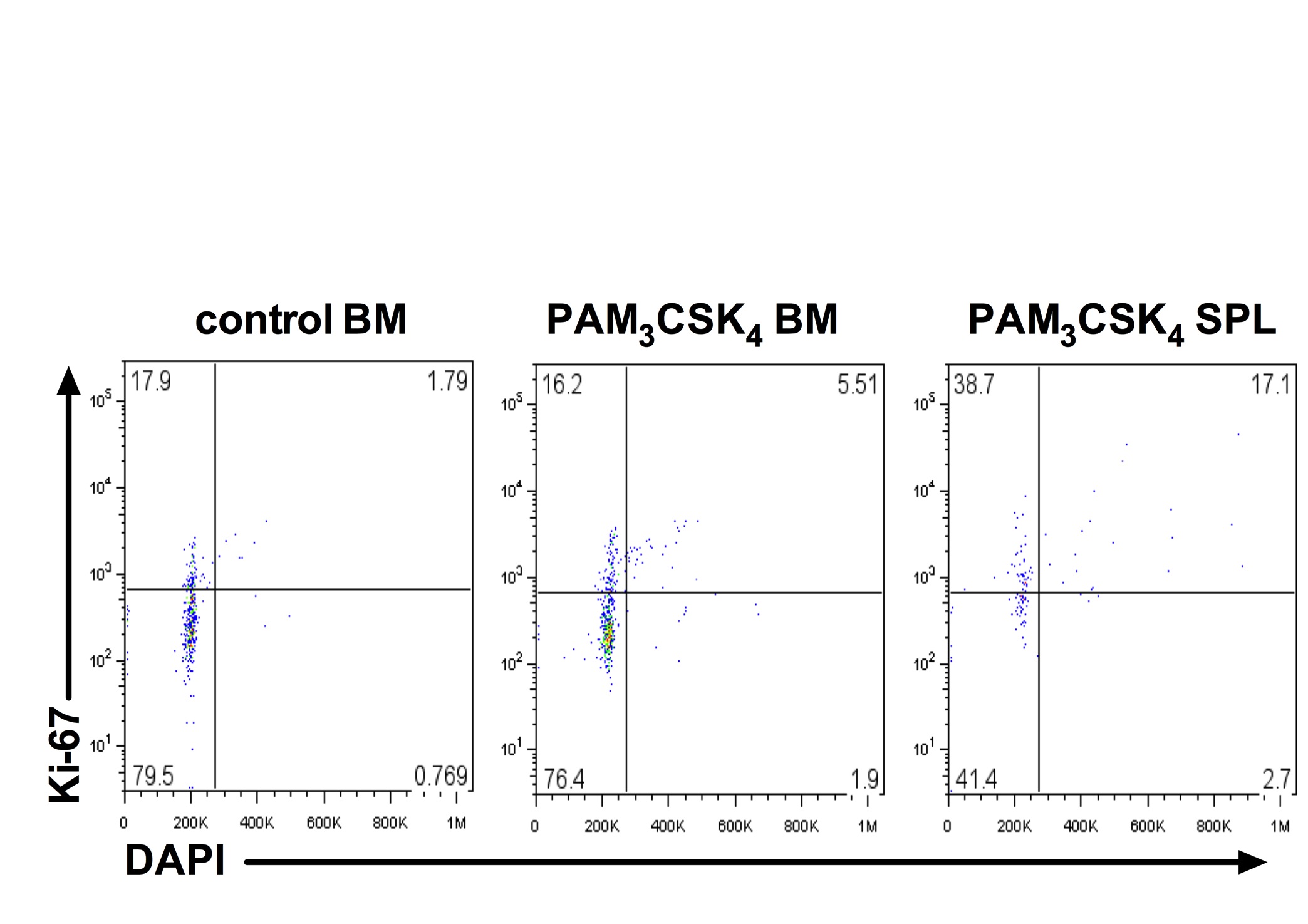


**Supplementary Figure 2**. **PAM_3_CSK_4_ treatment leads to increased cycling of HSCs in the spleen.** Wild-type mice were treated with PAM_3_CSK_4_ (100 µg/dose x 3 doses IP spaced 48 hours apart, analyzed 24 hours after the final dose) or water alone. Shown are representative flow plots of KSL SLAM cells from the bone marrow of a water control-treated mouse and the bone marrow and spleen of a PAM_3_CSK_4_-treated mouse.


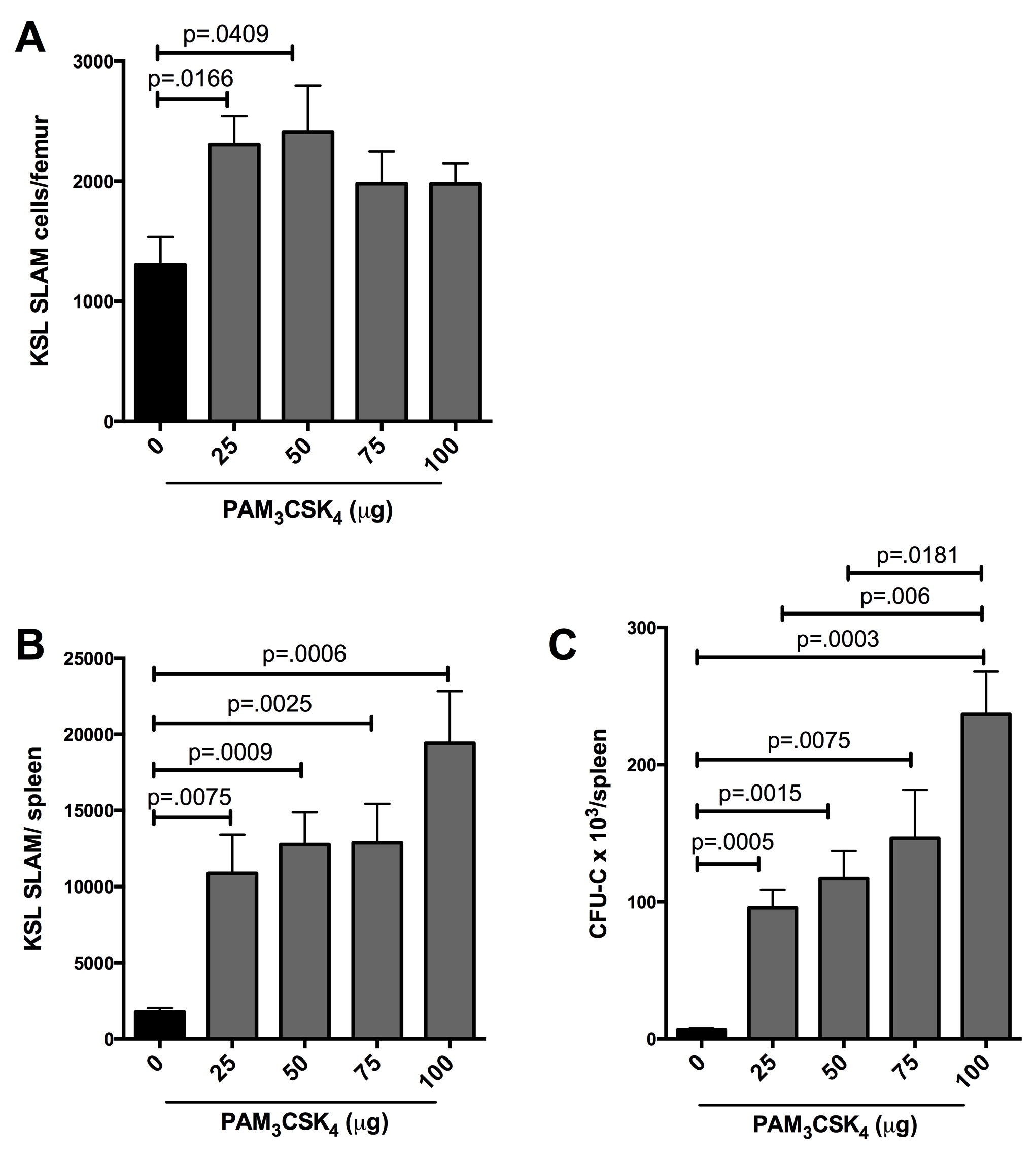


**Supplementary Figure 3. Dose-dependent effects of PAM_3_CSK_4_ on HSPC expansion.** Wild-type mice (6-8 weeks old) were treated with 0-100µg of PAM_3_CSK_4_ (3 doses IP spaced 48 hours apart, analyzed 24 hours after the final dose), and bone marrow and spleens were harvested for enumeration by flow cytometry of KSL SLAM cells in the femur (**A**) and spleen (**B**). In addition, whole spleen cells were plated in complete methylcellulose and colony formation was scored after 7 days of growth at 37C (**C**). N= 4-5 mice per dose level. Error bars represent mean +/- SEM. p values were determined by two-tailed Student’s t-test.

**
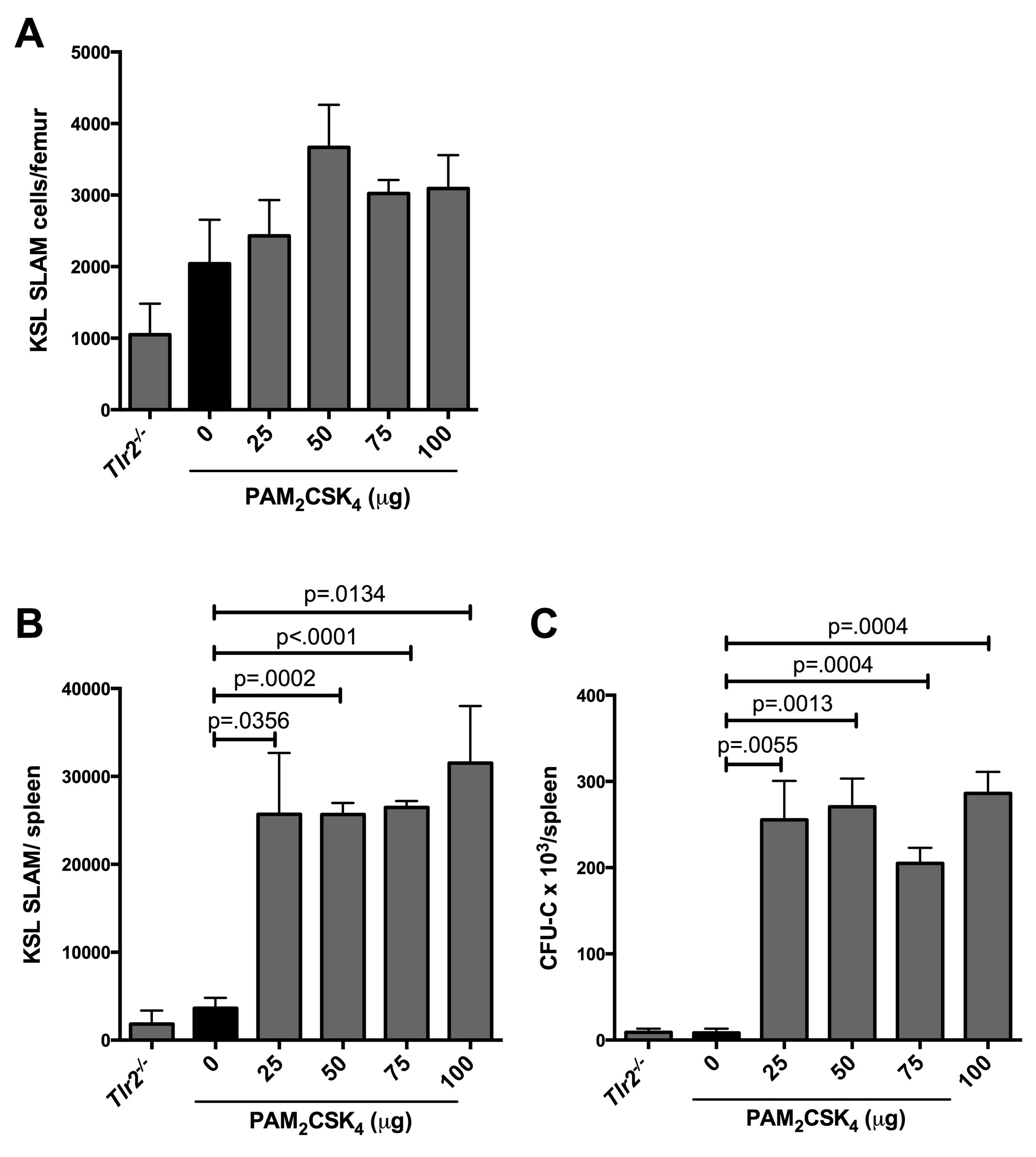
Supplementary Figure 4. PAM_2_CSK_4_ expands bone marrow and spleen HSPCs.** Wild-type mice (6-8 weeks old) were treated with 0-100µg of the TLR2/6 agonist PAM_2_CSK_4_ (3 doses IP spaced 48 hours apart, analyzed 24 hours after the final dose), and bone marrow and spleens were harvested for enumeration by flow cytometry of KSL SLAM cells in the femur (**A**) and spleen (**B**). In addition, whole spleen cells were plated in complete methylcellulose and colony formation was scored after 7 days of growth at 37C (**C**). *Tlr2^-/-^* mice were treated simultaneously as controls; they received the 100µg dose. N= 3 mice per dose level. Error bars represent mean +/- SEM. p values were determined by two-tailed Student’s t-test.


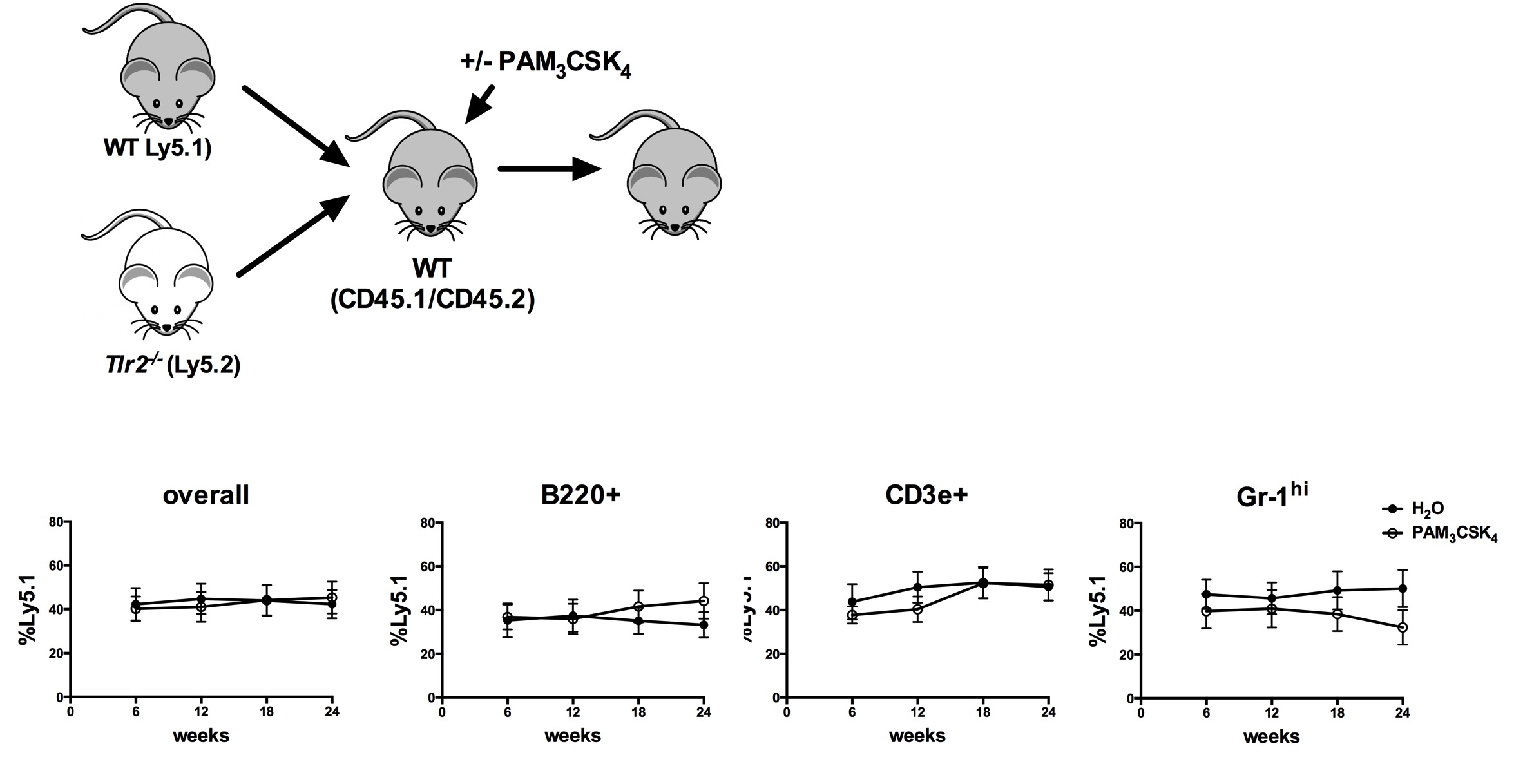


**Supplementary Figure 5. TLR2 agonist effects on HSPCs are, at least in part, cell non-automomous.** Chimeric animals were generated by transplanting equal numbers of wild-type (CD45.1) and *Tlr2^-/-^*(CD45.2) bone marrow cells into lethally irradiated wild-type (CD45.1/CD45.2) recipients. After allowing time for engraftment (12 weeks), recipients were treated with PAM_3_CSK_4_ (100 µg IP q48 hours x 3 doses). Bone marrow from treated chimeras was then transplanted into new lethally irradiated wild-type recipients, and the frequency of wild-type (CD45.1) donor cells, including overall leukocytes, B cells (B220+), T cells (CD3e+) and neutrophils (Gr-1^hi^) was determined in the peripheral blood of the recipients over time. Data represent 13-14 mice per treatment group from 3 independent transplants. Error bars represent mean +/- SEM.

**
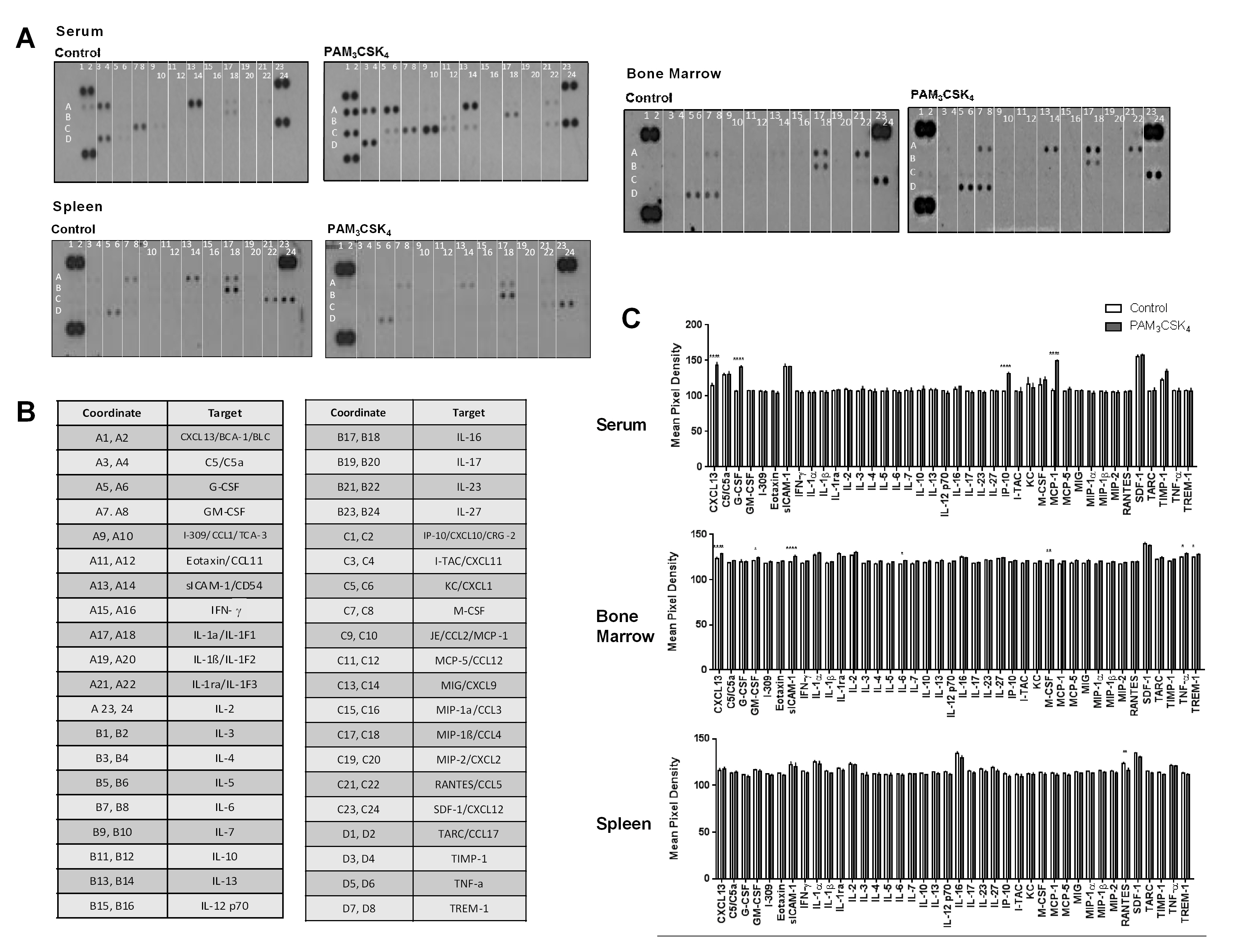
**

**Supplementary Figure 6. Cytokine expression following TLR2 agonist treatment.** Wild-type mice (6-8 weeks old) were treated with PAM_3_CSK_4_ (100 µg IP) or water control, and cytokine expression was measured in the serum, bone marrow, and spleen 16 hours later. (**A**) Shown are representative cytokine array blots from the serum, bone marrow and spleen, as indicated, of PAM_3_CSK_4_ or water control treated mice, with the legend depicted in panel (**B**). Membranes were exposed to X-ray film, and signal intensities were quantified for each cytokine in each tissue as summarized in (**C**). Data represent the mean from each treatment group from 2 separate experiments. Statistical significance was assessed using a two-way analysis of variance (ANOVA) followed by Sidak’s multiple comparisons test. *p<0.05; **p<0.01; ****p<0.0001.

**
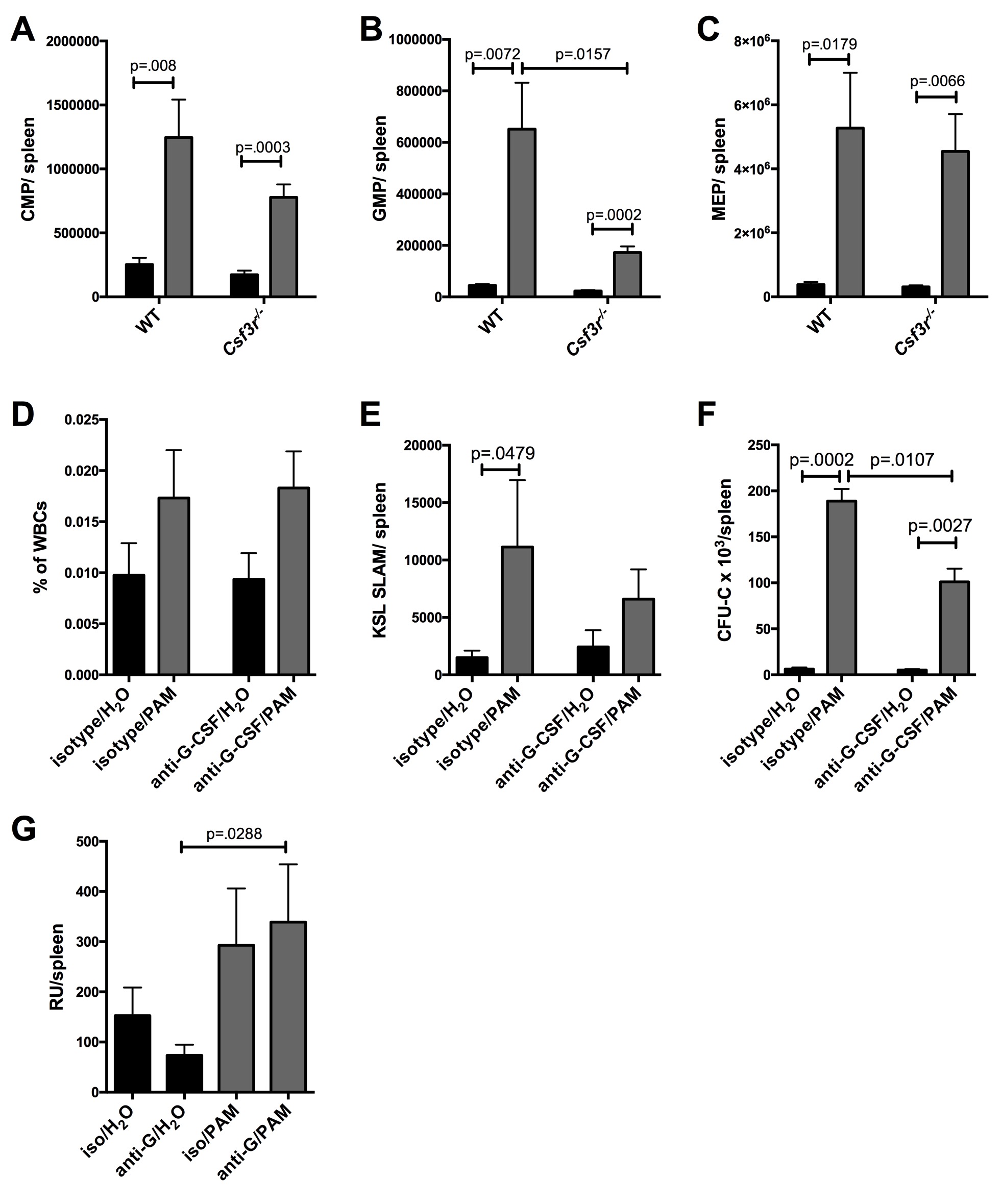
**

**Supplementary Figure 7. Inhibition of G-CSF partially mitigates the expansion of HSPCs upon PAM_3_CSK_4_ exposure.** WT or *Csf3r^-/-^* mice (6-8 weeks old) were treated with PAM_3_CSK_4_ (100 µg IP q48 hours x 3 doses, analyzed 24 hrs after final dose), and spleen myeloid progenitor numbers were enumerated by flow cytometry; shown are the absolute numbers of CMPs (**A**), GMPs (**B**) and MEPs (**C**) per spleen (n= 6-8 mice per group). Next, WT mice

were similarly treated with PAM_3_CSK_4_ or water alone and either a G-CSF neutralizing antibody or isotype control. Shown are the frequency of KSL SLAM cells in the bone marrow (**D**) and the absolute number of KSL SLAM cells in the spleen (**E**). (**F**) Whole spleen cells were plated in complete methylcellulose and myeloid colony formation was scored after 7 days of growth at 37C. N=3 mice per group. (**G**) To determine spleen HSC function, 2 x 10^6^ whole spleen cells from control water-treated or PAM_3_CSK_4_-treated mice that were also treated with a G-CSF neutralizing antibody or isotype control were transplanted along with 1 x 10^6^ whole bone marrow competitor cells into lethally irradiated wild-type recipients. Shown are total repopulating units per spleen. Data represent 9-10 mice per group from 2 independent transplants. Error bars represent mean +/- SEM. p values were determined by two-tailed Student’s t-test.


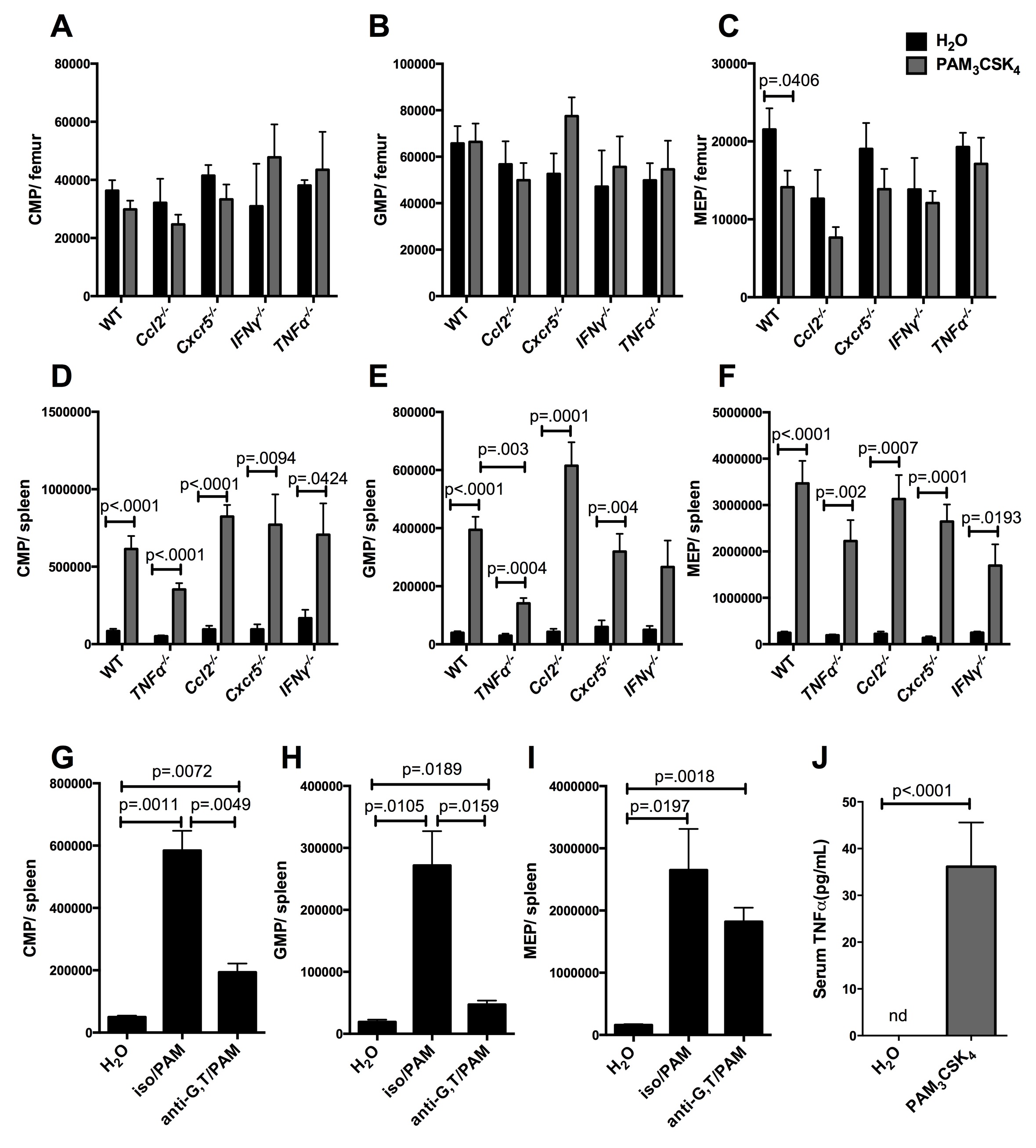


**Supplementary Figure 8. TNF***α* **contributes to the PAM_3_CSK_4_ mediated effects on HSPCs.** WT, *TNFα*^-/-^, *Ccl2*^-/-^, *Cxcr5*^-/-^, and *IFNγ*^-/-^ mice (6-8 weeks old) were treated with PAM_3_CSK_4_ (100 µg IP q48 hours x 3 doses, analyzed 24 hrs after final dose) or water alone. Shown are the GMPs, CMP, and MEPs per femur (**A-C**) and spleen (**D-F**) (n=4-12 mice per group). (**G-I**) WT mice were treated with PAM_3_CSK_4_ as described above, and in addition some mice received G-CSF and TNFα-neutralizing antibodies or isotype control antibody prior to PAM_3_CSK_4_ injections. Shown are the CMPs (**G**), GMPs (**H**), and MEPs (**I**) in the spleen as determined by flow cytometry for each treatment group (n= 3 mice per group). (**J**) WT mice were given a single IP injection of PAM_3_CSK_4_ (100ug), and serum TNFα levels were determined by ELISA 8 hours later (n=8 mice per group). Error bars represent mean +/- SEM. p values were determined by two-tailed Student’s t-test.
